# Implications of leaky gut in cattle

**DOI:** 10.1093/af/vfag012

**Published:** 2026-04-03

**Authors:** M Victoria Sanz-Fernandez, Juliette Wilms

**Affiliations:** Trouw Nutrition R&D, Amersfoort, 3800 AG, The Netherlands; Trouw Nutrition R&D, Amersfoort, 3800 AG, The Netherlands

**Keywords:** cattle health, cattle performance, gastrointestinal health, gastrointestinal permeability

Implications

- Leaky gut may contribute to systemic inflammation and disease in cattle, but its actual impact is likely to be context-dependent and influenced by multiple factors.
- Measurements of gastrointestinal permeability should not be interpreted in isolation, as increased permeability can occur alongside positive health and performance outcomes.
- When researching and designing nutritional solutions, supporting overall gastrointestinal health is probably a more effective goal than just focusing on reducing gastrointestinal permeability.

## Introduction

In recent years, leaky gut has emerged as a common denominator for seemingly unrelated challenges experienced by farm animals. Conditions such as heat stress, infectious diseases, feed restriction, and psychological stress have all been linked to disruptions in the gastrointestinal (**GI**) barrier function (Horst et al., 2025). However, the relative contribution of the GI tract, and more specifically leaky gut, to the systemic consequences of such challenges remains poorly understood.

A limitation in this area of research is the loose use of definitions. Terms such as gut health and integrity, barrier function, and intestinal permeability are frequently interchanged. Intestinal permeability is a measurable characteristic of the GI barrier function, which represents the physical, chemical, and immune separation between the gut lumen and the host (Bischoff et al., 2014). Leaky gut can be defined as an increase in permeability due to functional impairment. In this review, when ­discussing leaky gut, we refer to changes in permeability along the entire GI tract, not only the small and large intestines. Nevertheless, the barrier function is a multilayered system capable of compensatory responses. As an example, mice overexpressing claudin-2, a tight junction protein, exhibit an increase in colonic permeability yet display resistance to experimentally induced colitis, a phenomenon attributed to an attenuated immune response (Ahmad et al., 2017). Thus, although measurements of GI permeability can provide insight into the pathophysiology of certain conditions, we should be careful when interpreting their results in isolation, as it is likely that the context determines its biological relevance (Ahmad et al., 2017).

In addition, it is technically difficult to isolate the direct effects of increased GI permeability on the health and performance of livestock, given that conditions in which leaky gut is thought to play a role typically affect not only other aspects of the barrier function but also other organs and systems. Ultimately, it is still to be demonstrated that restoring the barrier function translates into improvements on GI or systemic health (Camilleri, 2019).

In this review, we aim to summarize challenges in cattle in which leaky gut appears to play a predominant role, while also presenting examples where GI permeability and phenotypic outcomes do not correlate. We will also explore and speculate on potential advantages of increased permeability and conclude with our perspective on the role of GI permeability measurements in animal nutrition research.

## Negative Consequences of Leaky Gut

One of the best examples of the direct consequences of leaky gut is the incidence of liver abscesses in beef cattle. According to the acidosis–rumentis–liver abscess theory, aggressive grain feeding can lead to rumen acidosis and damage to the rumen wall, providing entry to *F. Necrophorum*, the most frequent pathogen isolated from the abscesses (Broadway et al., 2024). There is a large variability in the incidence of liver abscesses in feedlots ranging between 10% and 30%, although these numbers widely vary among publications (Sanz-Fernandez et al., 2020). Further, multiple factors seem to play a role in this disorder, including nutrition, management practices, breed, and geographical location (Sanz-Fernandez et al., 2020; Broadway et al., 2024). Based on this multifactorial nature, it is unlikely that the development of liver abscesses represents purely an increased GI permeability issue. Rather, it appears that whether abscesses develop or not is context-dependent and modulated by factors inside and outside the GI tract.

On the dairy side, it has been hypothesized that leaky gut may contribute to the pathophysiology of metabolic diseases typical of the transition period. Stressors known to induce GI barrier dysfunction, like the abrupt change to a highly fermentable diet, the decrease in voluntary intake, and social and psychological stress, occur simultaneously around parturition (Sanz-Fernandez et al., 2020). At the same time, excessive systemic inflammation during this period has been associated with an increased incidence of diseases like ketosis, hypocalcemia, and fatty liver, with some authors proposing a causative role for inflammation itself (Horst et al., 2021). While the uterus and the mammary gland are obvious contributors to systemic inflammation around calving, the GI tract may also play a role (Bradford et al., 2015). In a model of intestinal barrier dysfunction in lactating cows, Kvidera et al. (2017) demonstrated that primary leaky gut elicited a systemic inflammatory response, evidenced by increased circulating inflammatory markers, anorexia, and decreased milk production. This remains one of the few studies in cattle showing a direct link between barrier dysfunction in isolation and systemic inflammation induction, with minimal confounding factors. Whether leaky gut is a relevant source of inflammation during the transition period remains to be elucidated.

**Figure 1.**
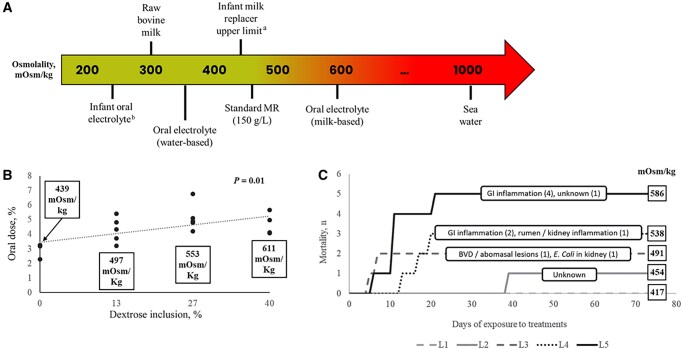
Increased osmolality in liquid feed induces leaky gut in calves. (A) Osmolality scale. (B) Twenty-four hours Cr-EDTA urinary recovery (as % of oral dose) in 7-wk-old calves. Treatments corresponded to milk replacers with different proportions of dextrose inclusion in exchange of lactose (adapted from Wilms et al. [2019]). (C) Schematic representation of time and cause of death in calves in response to milk replacers with different proportions of glucose inclusion in exchange of lactose: L1 = 0%, L2 = 10%, L3 = 20%, L4 = 30%, L5 = 40% (adapted from Wilms et al. [2020a]). ^a^According to the American Association of Pediatricians. ^b^According to the World Health Organization.

Looking into calf rearing, high osmolality of liquid feed is a well-documented challenge that results in health issues derived from the subsequent GI barrier dysfunction. Hypertonic solutions have been shown to increase paracellular permeability in calves and other species (Wilms et al., 2019). The physiological osmolality of bovine whole milk is approximately 300–350 mOsm/kg (Wilms et al., 2024a; Figure 1A), a range that supports efficient nutrient absorption and preserves gut health. In contrast, milk replacers (**MR**) often contain elevated lactose concentrations (40% to 50% DM), more minerals, and higher total solids (15% to 20% DM), pushing osmolality above 450 mOsm/kg (Wilms et al., 2020a), and sometimes over 600 mOsm/kg when electrolyte solutions are mixed directly into the milk (Wilms et al., 2020b). Because plasma osmolality is around 285 mOsm/kg, fluids with osmolality near 600 mOsm/kg exert a strong osmotic gradient across the gut epithelium, pulling water from the interstice and causing structural damage to the junctional complex (Cronje, 2007). As a result, hyperosmolar stress affects calf health by inducing diarrhea (Sweeny et al., 1974). This has been demonstrated in a series of studies performed by our group in which milk replacer osmolality was linearly increased by exchanging lactose with dextrose. Increasing milk replacer osmolality resulted in a linear increase in gastrointestinal permeability, as determined by greater urinary recovery of markers, such as lactulose, D-mannitol, and Cr-EDTA in calves (Wilms et al., 2019; Figure 1B). Furthermore, mortality also linearly increased with gastrointestinal damage being prevalent in the postmortem evaluation (Wilms et al., 2020a; Figure 1C). Nevertheless, the involvement of other factors like nutrient composition (e.g., increased glucose content) cannot be ruled out.

## The Importance of Context

In line with the above-mentioned examples, leaky gut has often been assumed to be consistently associated with undesirable ­phenotypic outcomes. However, as GI permeability techniques are increasingly applied in cattle studies across diverse conditions, emerging evidence suggests that this association may not always hold true. For instance, Silva et al. (2023) reported increased urinary recovery of a permeability marker (i.e., Cr-EDTA) in newly received feedlot cattle supplemented with a liquid formulation post-transportation, compared to those receiving a dry supplement. Unexpectedly, heifers in the liquid supplement group exhibited a more favorable phenotype, characterized by higher dry matter intake and decreased circulating inflammatory markers, suggesting reduced systemic inflammation.

Similarly, the proportion of dietary fat inclusion in MR can also modulate gut permeability in calves (Amado et al., 2019), though its effects are sometimes counterintuitive. Increasing fat (i.e., fat content >20% DM) and reducing lactose better aligns with the macronutrient profile of bovine whole milk (fat content ∼30% DM). It also lowers MR osmolality, which has been shown to improve fecal scores in calves (Amado et al., 2019), to reduce the need for medication (Berends et al., 2020), and to increase gut weight (Welboren et al., 2021). However, these studies also report increased intestinal permeability in calves fed high fat MR, which may not necessarily reflect a pathological deterioration of the gut barrier but rather a physiological consequence of a higher lipid absorption.

In the context of human obesity, an increased fat intake can lead to intestine-derived LPS being incorporated into micelles and chylomicrons (due to its insoluble component lipid A) and absorbed along with lipids (Boutagy et al., 2016). As a result, circulating LPS concentrations may appear elevated, even though tight junction integrity is not structurally compromised. Whether a similar process affects the absorption of the GI permeability markers (e.g., Cr-EDTA) is currently unknown. In addition to increased permeability, calves fed high-fat MR showed downregulation of genes related to tight junctions and upregulation of genes involved in epithelial transport and lipid metabolism (Welboren et al., 2021). In view of the apparent beneficial effects of high fat MR on health, we speculate that these changes at the molecular level indicate adaptations of the intestinal barrier to higher lipid flux. It should be noted that the fatty acid profile of MR differs from that of bovine whole milk fat, as alternative vegetable or animal fat sources are typically used (Wilms et al., 2024b). Such differences could influence GI barrier function; however, we do not observe a significant effect of the fatty acid profile on gut permeability in calves (Wilms et al., 2024a). Therefore, elevated permeability in calves fed high-fat MR should be interpreted with caution as it may represent metabolic signaling and absorptive adaptations rather than GI barrier dysfunction.

**Figure 2.**
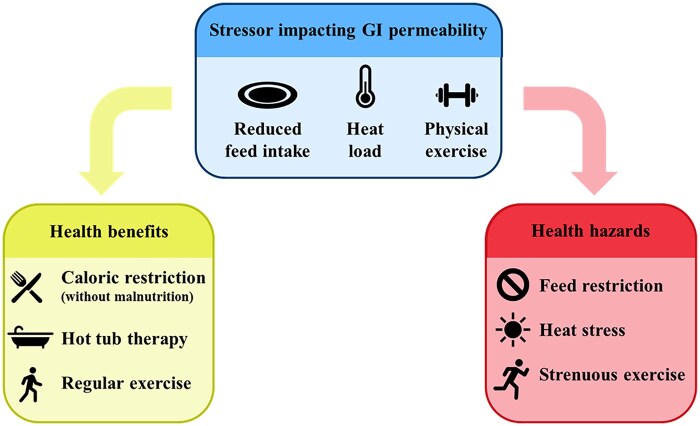
The consequences of leaky gut on health are context dependent. Stressors known to increase intestinal permeability can ultimately be beneficial or detrimental for health. Factors like the type of stressors, its magnitude and duration, and the presence of stacking stressors are all likely to modulate the overall response.

Not only are changes in intestinal permeability sometimes associated with unanticipated benefits but also stressors known to impair barrier function have also been linked to both beneficial and detrimental health outcomes (Figure 2). Conditions such as feed restriction, heat stress, and high-intensity exercise are consistently shown to compromise GI barrier integrity, which is thought to partially mediate their negative effects on health and performance (Chantler et al., 2021; Horst et al., 2025). Conversely, stressors similar in nature like fasting and caloric restriction, hot tub therapy, and regular exercise have been reported to have overall health benefits, although their consequences on GI health are largely unknown (Von Schulze et al., 2020; Green et al., 2022). Ultimately, whether increased GI permeability is harmful or not likely depends on the specific context. The nature of the challenge, its magnitude and duration, and the presence of stacking stressors are all likely to modulate the overall response.

## Harnessing the Benefits of Leaky Gut?

As described in the previous section, although increased GI permeability is expected to elicit negative responses, there are instances in which GI barrier function measurements and phenotypic outcome do not correlate. Moreover, we can reflect on scenarios in which reducing GI permeability might be detrimental. Although technically not leaky gut, the absorption of immunoglobulins (**Ig**) from colostrum in the first 48 h of the life of calves represents a situation in which absorptive capacity is physiologically increased. Immunoglobulin absorption is critical for the acquisition of passive immunity in animals that are otherwise born with little adaptive immunity. This process is extremely time sensitive as maturation of the enterocytes makes them lose this capacity (Hiltz and Laarman, 2019). The importance of not interfering with this process is illustrated by studies on dietary butyrate supplementation. While a substantial body of research supports the benefits of incorporating butyrate into MR, promoting GI development and improving health and growth outcomes (Górka et al., 2018); its inclusion in colostrum has been shown to reduce IgG absorption (Hiltz and Laarman, 2019). This is likely due to an acceleration in the maturation process of enterocytes, making them lose their absorptive capacity (Hiltz and Laarman, 2019) and likely explains why colostrum’s butyrate content is low, as compared to mature milk. Furthermore, these results underscore the need to carefully evaluate strategies that potentially result in decreased intestinal permeability by considering the physiological context.

Additionally, it is worth speculating whether certain aspects of increased GI permeability could be harnessed for benefit. The concept of hormesis refers to the adaptive or protective responses elicited by organisms when exposed to low doses of certain stressors that, at higher doses, would be harmful (Bauer et al., 2024). This concept has been frequently used to explain the beneficial effects of dietary restriction without malnutrition on health and longevity in various mammalian models (Green et al., 2022). Similarly, heat treatments through hot tubs and saunas have consistently shown a positive effect on metabolic health and aging-related disorders (Von Schulze et al., 2020). In both cases, a low dose of the corresponding stressor induces health benefits through acclimation, while feed restriction with malnutrition or an excessive heat load leading to heat exhaustion or heat stroke would severely compromise health.

Interestingly, GI permeability appears to adapt over time in response to certain stressors. For example, the increase in GI permeability observed in dairy cows during acute exposure to feed restriction (60% feed restriction; Horst et al., 2020) and heat stress (Fontoura et al., 2022) was no longer evident after 5 and 13 d of continued exposure, respectively. This implies the triggering of mechanisms that ultimately allowed the restoration of the barrier function despite ongoing stress. In a study by Rodriguez-Jimenez et al. (2025), intestinal permeability measured during recovery from heat stress was not only reduced relative to the stress period but also fell below pre-stressor baseline values. These findings suggest the activation of compensatory mechanisms that remain in place once the stressor is removed. How long these changes prevail over time is currently unknown. Overall, this evidence opens the possibility that controlled, low-dose stressors could be strategically harnessed to enhance intestinal barrier resilience through hormetic adaptation.

## Future Perspectives and Concluding Remarks

Gastrointestinal permeability measurements are increasingly being incorporated into livestock research, contributing to the development of this field. However, it is important to acknowledge that our understanding in this area is still incipient. First, it is critical to recognize that permeability represents a partial measurement of the GI barrier function, the latter being a complex multilayer system capable of compensating at different levels. This complexity might partially explain situations where an increase in permeability is detected in response to interventions that otherwise elicit positive effects on overall animal health and performance. It also highlights the importance of avoiding interpreting the results of GI permeability tests in isolation and the need for multiple measurements to appropriately evaluate the GI barrier function. Thus, the authors recommend incorporating complementary analyses, such as tissue histological assessments and the evaluation of tight junctions through gene and protein expression, as well as immunohistochemical techniques. Moreover, the biological relevance of changes in GI barrier function should be always investigated as they are likely to be context dependent. This is of particular relevance when it comes to GI permeability measurements in response to nutritional interventions. In the authors’ opinion, studies where intestinal permeability is assessed, which are likely to involve small numbers of animals due to technical difficulties intrinsic to the tests, should be complemented by studies with larger animal numbers able to capture changes in performance, overall health, reproduction, etc. This comprehensive approach is essential to avoid misinterpretation of the impact of nutritional interventions solely based on their ability to modulate permeability.

Ultimately, while reducing leaky gut is theoretically expected to benefit GI and overall health in animals, a direct cause-and-effect relationship has yet to be established. From a practical standpoint, aiming at a broader goal to support overall GI health, rather than focusing solely on permeability, is likely a more effective strategy for enhancing health and performance in cattle.

## About the Authors

**M. Victoria Sanz-Fernandez** is a ruminant researcher at Trouw Nutrition R&D. She holds a veterinary degree from Universidad Complutense de Madrid (Spain) and a PhD in Nutritional Sciences from Iowa State University (USA). Her current research focuses on gastrointestinal health in cattle and its implications for overall health and performance.

**Juliette Wilms** is a ruminant researcher at Trouw Nutrition R&D. She holds an MSc in Animal Science from AgroParisTech (University of Paris-Saclay, France) and a PhD from the University of Guelph (Canada). Since joining Nutreco in 2015, her research has focused on calf nutrition and health, with a particular emphasis on milk replacer composition and oral rehydration therapy.
